# Chronic Heart Failure in Children: State of the Art and New Perspectives

**DOI:** 10.3390/jcm12072611

**Published:** 2023-03-30

**Authors:** Biagio Castaldi, Elena Cuppini, Jennifer Fumanelli, Angela Di Candia, Jolanda Sabatino, Domenico Sirico, Vladimiro Vida, Massimo Padalino, Giovanni Di Salvo

**Affiliations:** 1 Pediatric Cardiology Unit, Department of Women’s and Children’s Health, University of Padua, 35122 Padova, Italy; 2 Pediatric Research Institute (IRP) Città della Speranza, University of Padua, 35122 Padova, Italy; 3 Pediatric Cardiac Surgery Unit, Department of Cardio Thoracic Sciences, University of Padua, 35122 Padova, Italy

**Keywords:** heart failure, pediatric heart failure, pharmacotherapy for heart failure, device therapy for chronic heart failure, pulmonary artery banding

## Abstract

Pediatric heart failure (HF) is an important clinical condition with high morbidity and mortality. Compared to adults, pediatric HF shows different etiologies characterized by different physiology, a different clinical course, and deeply different therapeutic approaches. In the last few years, new drugs have been developed and new therapeutic strategies have been proposed with the goal of identifying an individualized treatment regimen. The aim of this article is to review the new potential drugs and non-pharmacological therapies for pediatric heart failure in children.

## 1. Background

Heart failure is a complex and progressive clinical and pathophysiological syndrome that results from any structural or functional impairment of ventricular filling or ejection of blood [1].

Chronic heart failure (HF) is classified into two types: HFrEF—heart failure with reduced ejection fraction; and HFpEF—heart failure with “preserved” EF. 

HFrEF occurs with inadequate ventricular contraction and is commonly associated with a dilated left ventricle. HFpEF primarily depends on diastolic dysfunction and increased ventricular filling pressures, leading to elevations in atrial and venous pressures and, in the case of LV dysfunction, an increase in pulmonary arterial pressure. Right heart failure (RVF) is not common in children but could be associated with several congenital heart diseases (CHD), including Tetralogy of Fallot, transposition of great arteries, atrial septal defects, Ebstein anomaly, arrhythmogenic right ventricular cardiomyopathy (ARVCM) and right ventricle dysfunction in single ventricle physiology [2]. The great variability of causes and different pathophysiological mechanisms generally lead to the development of the same symptoms: jugular venous distention, hepatomegaly, and occasionally peripheral edema and ascites [3]. 

Pediatric HF differs from adult HF, mainly due to the variety of etiologies classified into three main categories: heart failure due to cardiomyopathy, heart failure due to CHD with biventricular or univentricular physiology, and heart failure from acquired heart disease [4]. The prevalence of CHD is around 0.8% of live births, and about 70% of them are at risk of HF. Fortunately, surgical or percutaneous treatment can effectively resolve or prevent the development of heart failure in those patients. Despite all, few papers are focused on the epidemiology of HF in CHD; the prevalence of CHD in severe HF ranged between 25 and 75%, with large geographical variations. Congenital heart diseases, such as an anomalous left coronary artery from the pulmonary artery (ALCAPA), critical aortic stenosis, coarctation of the aorta, and single-ventricle physiology, are most frequently associated with HF in newborns and infants [5]. Dilated cardiomyopathy (DCM) is a leading cause of HF in older children and is one of the main indications for pediatric heart transplantations. The incidence is 0.58–0.73 per 1,000,000 children/year; it is more common in adolescents, with an incidence of 4.4 to 4.8 per 100,000 [6] (Table 1). 

The diagnosis of HF is an integration of clinical signs, symptoms, and instrumental investigations. A chest X-ray and EKG can help determine whether nonspecific signs such as tachypnea, dyspnea, and failure to thrive are attributable to heart failure. On the chest radiograph, the cardiac shadow may be enlarged, and in some cases, signs of increased pulmonary vascular texture, alveolar edema, and pleural effusion may be noted. The EKG commonly shows nonspecific abnormalities such as ventricular hypertrophy or repolarization abnormalities, or some patients can present a rhythm alteration (supraventricular tachycardia, atrial fibrillation, atrial flutter, atrioventricular block, and ventricular tachycardia). Laboratory markers are a useful tool to establish the diagnosis of HF. The most important are BNP (atrial natriuretic peptide) and pro-BNP (N-terminal protein of BNP); those markers are often elevated in children with HF and may represent poor prognostic factors [7]. It has demonstrated a strong correlation between increased NT-pro-BNP and the worst symptoms and decreased ventricular function [8]. Electrolytes and renal function may be impaired, especially in patients with acute decompensation. Hyponatremia is frequently identified in patients with advanced disease and is associated with worse outcomes in hospitalized patients, which is why it is recommended to monitor renal function in these patients to prevent renal insufficiency [9]. Echocardiography remains the fundamental exam for the diagnosis of heart failure. In general, echocardiography allows for the evaluation of the dilatation of the cardiac chambers, the systolic or diastolic dysfunction, and any associated cardiac defects. It is mandatory to rule out coronary abnormalities in infants with a first finding of dilated cardiomyopathy to exclude ALCAPA [4]. 

Heart failure remains a challenge for pediatric cardiologists: most of the recommendations for chronic pediatric HF management were extrapolated from adult heart failure trials, despite the fact that the etiopathology of the two groups is different. Thus, the development of novel therapeutic approaches for the treatment of this disorder is crucial. In addition, few data are available on the epidemiology and clinical outcome of heart failure in the pediatric population, mostly focused on the hospitalization rate. A retrospective analysis showed that hospitalizations for cardiomyopathies and heart failure in children were significantly longer than in adults. The overall mortality rate was higher for pediatric patients [10], in particular when HF was associated with CHDs (>20% in this subgroup) [11].

This review discusses the current and future pharmacological and device therapies in children with chronic HF and the need for future clinical trials in children for the safety and efficacy of newer drugs that are used in adults. 

## 2. Medications 

In the last few decades, medical therapy has been based on the neurohormonal paradigm. The hypothesis assumes that the impairment of heart function drives the activation of the compensatory mechanisms, including the adrenergic nervous system, the renin-angiotensin system, and the cytokine system. The progressive activation of these systems leads to secondary end-organ damages that usually worsen symptoms and decompensate HF [12]. Thus, by blocking this mechanism’s pathway, it might be possible to prevent the shift to a decompensated HF and, finally, to reduce symptoms, hospitalizations, and deaths. In the last European and American guidelines [13], recommendations about medical therapy in chronic heart failure are based on whether the left ventricular ejection fraction is reduced or preserved. Based on several randomized clinical trials [14,15], diuretics, ACE inhibitors, mineralocorticoid antagonists, angiotensin receptor antagonists, and beta blockers are recommended in HFrEF patients. In HFpEF patients, no treatment has been shown to reduce mortality and morbidity; diuretics can be used to reduce symptoms of congestion. 

Unfortunately, in the pediatric population, there are no similar levels of evidence. Many obstacles must be overcome to perform pediatric HF studies, including heterogeneous etiologies of HF, complexity in building trials in pediatrics, deep changes in weight during growth, complexities of pediatric drug formulation, dose selection, comorbid conditions, a small number of patients eligible for these studies in general, and for single centers [16]. Thus, current pharmacological therapy for pediatric HF is limited and mostly based on adult studies. In Table 2, we provide the most common pediatric HF drugs and doses based on our clinical practice.

### New Drugs for Heart Failure Treatment

Ivabradine is an inhibitor of the funny channels located in the sinoatrial node that slows the rate of phase-4 depolarization, reducing the heart rate. These channels (or If, or IKf, also referred to as the pacemaker current) are directly modulated by cyclic nucleotides (such as cyclic AMP) and are the only ones that are activated in hyperpolarization and can generate the diastolic depolarization phase of the pacemaker potential and mediate autonomic control of cardiac rate [17]. It was demonstrated that elevated heart rates are associated with mortality in heart failure and are directly related to the risk of cardiovascular death or admission to the hospital in patients with heart failure [18]. This drug was studied in adult HF populations in the SHIFT and BEAUTIFUL studies [19]. The conclusion enlightened the importance of ivabradine to improve clinical outcomes in patients with LV dysfunction, despite the primary clinical presentation. Registry data from children with dilated cardiomyopathy demonstrated that an elevated heart rate was associated with an increased risk of death and cardiac transplant [20]. The safety of Ivabradine in children has been validated in a pediatric randomized, double-blind, placebo-controlled, phase II/III clinical trial of children with stable HF [21], which showed a reduction of the resting heart rate and an increase in the left ventricular ejection fraction. Ivabradine was approved by the FDA in 2019 for treatment in children with symptomatic heart failure >6 months of age.

Neprilysin is an enzyme whose main effect on the cardiovascular system is to degrade natriuretic peptides, which are helpful to decrease sympathetic tone and aldosterone levels, with the subsequentially effect of lowering blood pressure and promoting sodium excretion. The association of a Neprilysin inhibitor with an angiotensin II receptor antagonist provides simultaneous inhibition of Neprilysin and blockade of the renin-angiotensin-aldosterone (RAAS) system. The association available on the market is sacubitril/valsartan. In the largest study, PARDIGM-HF (2009–2014), more than 8000 patients received alternatively sacubitril/valsartan or enalapril. The sacubitril/valsartan group showed a reduction of 3.7% in the primary end point of death from cardiovascular causes or hospitalization for heart failure and a 3.2% reduction in overall mortality [22]. In the pediatric population, an international multicenter study, PANORAMA-HF, was designed to assess the pharmacokinetics (PK) and pharmacodynamics (PD) of sacubitril/valsartan and to evaluate the superiority of sacubitril/valsartan over enalapril for the treatment of pediatric HF. In October 2019, based on the data from the adult population and the partial data of the PANORAMA-HF trial, the FDA approved the use of sacubitril/valsartan for pediatric patients with symptomatic heart failure with left ventricular systolic dysfunction, one year of age and older. The trial was completed in January 2022 with 393 patients enrolled. It is now waiting for all subjects to complete 52 weeks of therapy before performing data analysis. 

SGLT2 inhibitors (canagliflozin, dapagliflozin, and empagliflozin) were historically designed for diabetics: the mechanism of action is inhibition of the sodium/glucose cotransporter situated in the kidney, to promote glucose excretion and improve glycemic control. Surprisingly, the trials showed a reduction in heart failure-related events in these patients. Recently, McMurray established in a multicenter randomized trial on 4744 patients with HFeEF that therapy with dapagliflozin reduces the risk of worsening heart failure or death from cardiovascular problems compared to a placebo, regardless of the presence of diabetes [23]. A second large trial (EMPEROR-Reduced) recently confirmed the results of Mc Murray et al., with the advantage that the benefits were already evident in the first 12 weeks [24]. Additionally, canagliflozin has been studied for HF. In the CHIEF-HF trial, 476 adult patients with HF were randomized to a canagliflozin arm or a placebo arm. After 12 weeks, patients show an improvement in quality of life as assessed by the KCCQ TSS without any differences in participants affected by type 2 diabetes or not [25]. Future studies should be addressed to compare the effectiveness of different SGLT2 inhibitors based on the avidity/selectivity of SGLT1 vs. SGLT2 receptors. 

In the first description of SGLT2 inhibitor use in pediatric HF, dapagliflozin was added to the HF therapy regimen in 38 patients (26 of them with HFrEF). The drug was well tolerated; no patients experienced symptomatic hypovolemia or hypoglycemia, and six patients experienced a symptomatic urinary tract infection requiring antibiotic treatment. In 26 patients with HFrEF, after 312 days of therapy, LVEF increased significantly from 32 to 37.2% [26]. 

Omecamtiv Mecarbil is the first drug working as a myotrope, improving myocardial function by directly augmenting cardiac sarcomere function with the goal of increasing systolic function in patients with HFrEF. In the GALACTIC-HF trial [27], 8256 adult patients with symptomatic chronic HFrEF (EF of 35% or less) were recruited to receive Omecamtiv Mecarbil or a placebo, in addition to standard heart failure therapy. The primary outcome was the time-to-first heart failure event or death due to cardiovascular causes. Omecamtiv Mecarbil significantly decreased the primary endpoint, especially in the group of patients with EF < 22%; there was a 16% reduction in the time-to-first heart failure event or cardiovascular death (HR: 0.84; 95% CI: 0.77–0.92; *p* = 0.0003). These findings suggest that patients with more severe heart failure have greater clinical benefits from cardiac myosin activator therapy. No data is currently available for children. 

Vericiguat, a novel oral soluble guanylate cyclase stimulator, an enzyme responsible for the production of nitrogen monoxide (NO), is involved in arteriolar vasodilation, the reduction of mechanical and oxidative vascular stress, and inhibiting fibrosis [28]. In a recent trial, VICTORIA, a number of 5050 adult patients with chronic symptomatic HF and an ejection fraction of <45% had a reduced risk of cardiovascular death, all-cause death, and HF hospitalization [29]. An interesting meta-analysis study compared the relative efficacy of sacubitril/valsartan, vericiguat, and SGLT2 inhibitors in patients with HFrEF and showed how the risk of HF hospitalization does not differ significantly between patients on SGLT2i or sacubitril/valsartan, while dapagliflozin is superior to vericiguat [30]. No data are currently available for children; however, a dedicated clinical trial about vericiguat in children’s HF is upcoming [31]. In Table 3, we summarize the clinical trials on new drugs for HF in pediatric and adult populations.

## 3. Interventional Cardiology

### 3.1. Device Therapy 

Device-based solutions to improve cardiac physiology in HF and to prevent or treat post-capillary pulmonary hypertension have been proposed in the adult population. For example, left ventricular expanders, mechanical circulatory support devices, and neurostimulators are used in the adult population, but there is no experience with this device in pediatric HF. The only option currently used in pediatric populations is an inter-atrial device. 

The creation of an inter-atrial shunt is advisable in several cardiovascular diseases, especially in HF. Increased left ventricular (LV) filling pressure and left atrial pressure are observed both in HFpEF and in HFrEF. Diminished relaxation can be due to a variety of causes, including secondary LV hypertrophy or restrictive cardiomyopathies. In addition, mitral valve regurgitation can further contribute to increasing LA pressure. A long-term increase in LA pressure determines progressive lung congestion and type II pulmonary hypertension. In addition, high LV filling pressures impact myocardial perfusion, rhythm abnormalities, and mechanical desynchrony. Thus, the creation of an interatrial shunt allows to reduce the left atrial pressure and left ventricular filling pressure. In addition, the LA shunt might reduce the LV volume overload, increasing the responsiveness to the pharmacologic therapy. Finally, RV overload might stiffen the interventricular septum and reduce septal dyskinesia. 

In the pediatric population, stent implantation into the interatrial septum or balloon dilatation of a pre-existing atrial communication or after a septal puncture has been used, especially in patients with complex heart anatomy, to improve symptoms [33]. These methods present some limitations: balloon dilatation often results in early reclosure and is thus effective in acute settings; in addition, the ASD size cannot be calibrated. A stent implantation is highly effective to create an ASD with a pre-fixed diameter; however, stent placement might be challenging, and the removal needs a surgical approach [34]. 

New inter-atrial devices, such as the Inter Atrial Shunt Device (IASD, Corvia Medical, Tewksbury, MA, USA), the V-Wave (Caesarea, Israel), and the Atrial Flow Regulator (AFR, Occlutech, Schaffhausen, Switzerland) interatrial shunt device, have been used to create a permanent controlled left-to-right shunting in adult patients [35,36]. Those devices have not been used in children yet, except for the AFR. 

**Figure 1:**
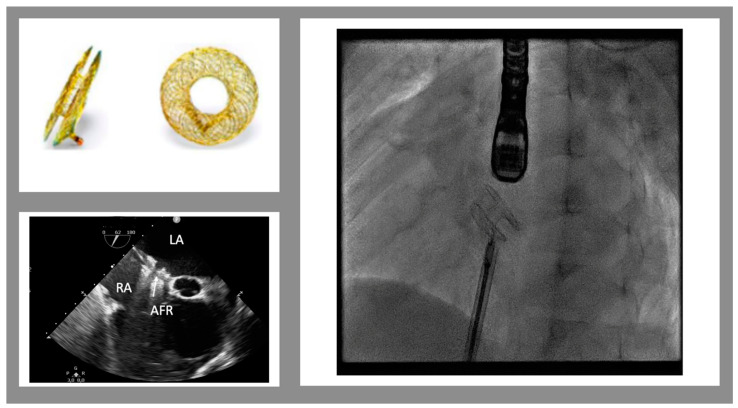
Atrial flow regulator device. Top on the left: the device in frontal and lateral views. The bottom on the left shows the trans-esophageal view of the implanted device (mid-esophageal view, 52°). On the right is a fluoroscopic view of the device just before the release. (LA left atrium, RA right atrium, AFR atrial flow regulator)

The AFR is a self-expandable, small implantable device made of Nitinol (Figure 1). It is similar to a self-centering atrial septal defect occluder device with a 4 to 10 mm hole in the middle (in Europe, only 8 and 10 mm obtained the CE mark). The device can be implanted, through a trans-venous approach, into an existing atrial septal defect/patent foramen ovale or after a trans-septal puncture and pre-dilatation with a 6 to 10 mm balloon catheter. The goal of this therapy is to maintain stable and pre-fixed interatrial communication in order to reduce the interatrial gradient to 5–8 mmHg. In clinical practice, reduced diastolic filling pressure leads to reduced symptoms, improved exercise tolerance, and a higher quality of life. 

The AFR is being evaluated for adult heart failure in two clinical studies (PRELIEVE and PROLONGER), in a prospective registry (AFteR Follow-up Study to Monitor the Efficacy and Safety of the Occlutech AFR in Heart Failure Patients), and in an ongoing clinical study for pulmonary arterial hypertension (PROPHET Pilot Study to Assess Safety and Efficacy of a Novel Atrial Flow Regulator in Patients With Pulmonary Hypertension). The prospective, non-randomized, multicenter, phase 2 PRELIEVE study reported the first-in-human use of the AFR for older patients with HFpEF (LVEF >40%; n 24) or HFrEF (LVEF 15–39%; n 29). The resting PAWP decreased by 5 mm Hg at 3 months after the AFR implantation. No complication occurred, in particular no shunt occlusion, stroke, or new right HF was observed during the 1-year follow-up period, with clinical improvements in certain patients [36,37]. 

In the pediatric population, some cases describe the safety and feasibility of the procedure in patients with PAH [38], Fontan’s failing circulation [39,40], and in young children [41]. In a recent multicenter US experience, AFR was implanted in six pediatric patients, five of whom had Fontan circulation failure and only one had left ventricular hypertension. All pediatric patients survived without complications. Oxygen saturations increased in all Fontan patients and were maintained through a follow-up of 8 weeks; also, NYHA class improved from a median of stage 3 before the procedure to a median of stage 2 after the procedure [39]. Currently, AFR in the pediatric population has been more frequently used for PAH and Fontan failure than heart failure compared to adults. However, a clinical case series with 3 patients (6 to 13 years old) affected by restrictive cardiomyopathy and undergoing AFR implantation to reduce LA volume overload and PAH postcapillary has been reported. The procedure improved LA dilation, postcapillary pulmonary hypertension, and HF symptoms in all 3 patients [42]. In another retrospective, single-center analysis, five pediatric patients with symptomatic heart failure not responding to maximal medical therapy and with a history of complex congenital or acquired cardiac diseases underwent AFR implantation. The procedure was successfully completed in all five patients. In one case, a hybrid procedure was performed during pulmonary artery surgical banding with a trans-atrial approach through the right atrial free wall. Clinical improvement was observed in all patients with a reduction of LA dilation, postcapillary pulmonary hypertension, and HF symptoms [43]. Follow-up studies are required to monitor the efficacy and safety of the Occlutech AFR and to identify the category of patients that can benefit most. 

### 3.2. Cardiac Resynchronization Therapy 

Cardiac resynchronization therapy is known worldwide (since 2009 American Heart Association guidelines on HF) to be useful for patients affected by HfrEF and LVEF < 35%. CRT is recommended in adult patients affected by HF with reduced ejection fraction (LVEF ≤ 35%), LV dilatation, and NHYA classes II, III, and IV despite maximization of drug therapy and duration of QRS > 120 ms. This therapy demonstrated a slower progression of heart failure due to the improvement of cardiac motion and a reduced risk of sudden death with a general improvement in quality of life [44].

According to the 2013 EHRA and AEPC pediatric guidelines, CRT might be used in pediatrics, especially for adolescents or young adults, with some clarifications. In pediatric and CHD populations, the QRS duration cut-off is the 98th percentile for age. In patients with specific progressive forms of DCM (ventricular non-compaction, neuromuscular, and mitochondrial disease), CRT should be considered carefully and individually because of the lack of evidence on efficacy. Patients with systemic ventricles could benefit from CRT, but with a specific evaluation of the mechanical dyssynchrony [45]. Some pediatric patients with a history of severe left ventricular dysfunction (LVEF < 30%) associated or not with ventricular arrhythmias secondary to CMD or CHD (valvular stenosis type aortic or mitral valve) may benefit from defibrillator-associated cardiac resynchronization therapy (CRT-D). 

## 4. Surgical Options

Surgical options were historically based on ventricular assist devices (VAD). VAD helps pump blood from the ventricle(s) to the rest of the body, and it could be used as a bridge-to-transplant. However, VADs have well-known limitations in pediatric patients, including high complication rates, such as bleeding, thrombosis, and infections, especially for infants with a body weight of less than 10 kg. Of note, pediatric heart availability for transplant is limited compared to adults, thus resulting in a potentially longer heart transplant waiting list. So, even though VADs have significantly reduced overall mortality in chronic heart failure patients, the pediatric heart transplant waiting list is still burdened by high morbidity and mortality.

### Pulmonary Artery Banding (PAB)

Historically, pulmonary artery banding (PAB) was a palliative procedure for patients with HF and a left-to-right shunt. Recently, some centers started to use PAB as a surgical option in selected infants or toddlers with dilated cardiomyopathy, as a bridge-to-recovery or -to-VAD or -to-transplant. The mechanism of PAB is perfectly summarized by Schranz et al. [46]. PAB-induced RV pressure overload straightens the interventricular septum, improving LV filling dynamics, LV end-diastolic volume, and pressure. The long-term impact on the LV involves a restored ventricular electromechanical synchrony, an increased LV ejection fraction, and less left atrial congestion. In conclusion, PAB allows clinical improvement and growth normalization with the improvement of the Ross Class of HF. The first-time application of PAB for the treatment of DCM was published in 2007 by Schranz et al. [47] in Giessen, reporting the case of a 2-month-old baby with dilated cardiomyopathy who recovered from end-stage heart failure and after pulmonary artery banding. A recent world network survey reported 70 patients from 15 centers in 11 countries from America, Asia, and Europe [48]. All the patients presented with end-stage left ventricular dilated cardiomyopathy with preserved right ventricular function. After PA banding, 60% of the patients showed a clinical improvement, and 48% showed a functional recovery in 1 year. 

The initial report from Germany was primarily focused on the use of PAB for cardiac recovery. Other case series have shown the potential utility of PAB as a tool for bridge-to-ventricular assist devices (VAD) or straight-to-heart transplants [46,49]. Several studies reported case series with their own inclusion/exclusion criteria. The best predictors for clinical response were age <1 year (usually age >3 years is an exclusion criterion), normal pulmonary artery pressure, and normal or mild left atrial dilatation. However, more studies should be conducted to identify the ideal candidate for PAB. Padalino et al. proposed a patient-tailored approach to end-stage heart failure in infants and children [50]. The treatment modalities were classified into 3 different situations: (1) patients <1 year with LV dysfunction due to DCM and preserved RV function were first considered for PAB according to the Giessen protocol. Exclusion criteria were: biventricular failure, moderate-to-severe tricuspid regurgitation, pulmonary hypertension, and associated major CHD. The PAB is reported to be tightened until right ventricular pressure increases to 70% of systemic pressure; (2) patients <10 years and <20 kg who did not meet PAB inclusion criteria were managed with either left or biventricular Berlin Heart; (3) patients >10 years or ≥20 kg with isolated LV failure underwent placement of intracorporeal Heartware (HVAD).

## 5. Nonpharmacological Interventions

In the HF Guidelines, a multidisciplinary approach is taken into consideration, which includes various medical and paramedical figures who contribute to creating a set of non-pharmacological therapies, including nutrition, physical activity, and mental health. Multidisciplinary teams facilitate the implementation of medical therapy, address potential barriers to self-care, reduce the risk of subsequent rehospitalization for HF, and improve survival. In pediatric HF, malnutrition is multifactorial and involves hypermetabolism, decreased intake, increased nutrient losses, inefficient utilization of nutrients, and malabsorption. It is well known that pediatric patients with heart diseases have a better post-operative outcome when surgery is performed in a setting of good nutritional status, and a low body mass index is a poor predictor of decreased graft survival after heart transplantation [51]. Emerging data suggest that CoenzymeQ10, a potent antioxidant, could improve cardiac function by stabilizing myocardial calcium-dependent ion channels and preventing the consumption of essential metabolites [52,53]. In children, only one study showed that ubiquinol supplementation of 10 mg/kg/die for 24 weeks could improve the ejection fractions and the NYHA class of 10 patients with HF in dilated cardiomyopathy [54]. Nutritional support, including supplementation and enteral and parenteral nutrition, should be considered an essential part of routine care. 

## 6. Future Perspectives

Novel clinical pharmacological trials specifically designed for the pediatric population are planned or ongoing. A new trial just started recruiting pediatric patients with HF due to systemic left ventricular dysfunction to assess the safety and efficacy of vericiguat in this population [30]. 

In the field of biomedical engineering, the miniaturization of VADs will make implantation possible even in smaller patients, reducing complications and recovery times [55]. In the genome editing field, CRISPR-Cas9 is a revolutionary system that permits the modification of specific sequences by insertion, deletion, or substitution using rationally designed templates. This technology has been tested using CHD animal models and has shown some promising results [56].

## 7. Discussion 

Heart failure in children remains a high mortality and morbidity disease. Compared to adults, pediatric HF shows different etiologies with deeply different therapeutic approaches. CHD is the most common cause of HF. Fortunately, in these patients, surgical or percutaneous CHD correction resolves symptoms and promotes the normalization of heart chamber volume and function. Myocarditis, genetic cardiomyopathies, and metabolic diseases are characterized by different physiologies, clinical courses, and responses to therapies. Thus, treatment standardization is very difficult. Finally, some specific disorders can be treated with targeted therapy (the use of supplements to replenish the affected deficiency in metabolic myopathies, such as the recombinant human acid alpha-glucosidase (rhGAA) enzymatic replacement therapy in Pompe’s disease) [57]. 

Irrespective of the availability of specific therapies, the pharmacological treatment of HF is intended to minimize symptoms, reduce the number of hospitalizations, and improve quality of life. Despite the lack of specific trials in pediatrics, diuretics, beta-blockers, ACE inhibitors, angiotensin receptor blockers, aldosterone antagonists, and digoxin remain the standard of care for chronic HF.

**Figure 2:**
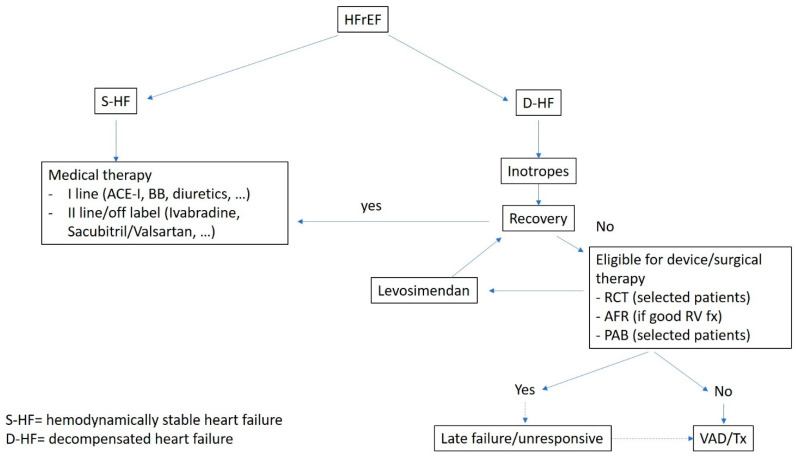
A suggested algorithm of treatment HFrEF.

A new approach in the adult world is device therapy. AFR is a new device designed to create stable and pre-fixed interatrial communication with a predetermined diameter. The aim of this therapy is to reduce LV and LA filling pressures to improve symptoms and quality of life. The effectiveness of this device was demonstrated in adults while no trial was started in pediatric patients. Some brief reports suggest that AFR might be considered to improve symptoms and reduce pulmonary pressure and pulmonary vascular resistances, such as in the bridge-to-transplant or bridge-to-transplantability approach. Differently from adults, in children has been demonstrated the possibility of cardiac remodeling, especially in the first years of age. In this setting, PA banding (getting straight the interventricular septum and reducing the LV end-diastolic diameter) may help the LV obtain an ellipsoidal shape, essential to a more efficient LV contraction pattern, and reduce the LV dyssynchrony. Unfortunately, this option is only applicable to young patients (<1–4 years) with preserved RV and tricuspid valve function. For all the other patients, VAD is the only option for end-stage HF. Our approach to HFrEF is summarized in Figure 2.

In conclusion, heart failure in children is a complex syndrome with heterogeneous etiology and clinical features and represents a great challenge for pediatric cardiologists. New options are available, and different strategies are possible. In the future, the development of child-specific data will be needed with the goal of identifying an individualized treatment regimen.

## Figures and Tables

**Table 1:** Main causes of pediatric heart failure.

Category	Diagnoses in Category
Congenital heart disease (CHD)	An anomalous left coronary artery from the pulmonary artery (ALCAPA), critical aortic stenosis, coarctation of the aorta, and single-ventricle congenital heart disease.
Inherited cardiomyopathy	HCM, RCM, DCM, ARVC, LVNC, fatty acid oxidation disorder mitochondrial disorders, Barth syndrome, Danon disease, and limb-girdle dystrophy.
Acquired conditions	Myocarditis, Kawasaki disease, arrhythmia, systemic lupus erythematosus, dermatomyositis, rheumatic heart disease, and chemotherapy.

HCM—hypertrophic cardiomyopathy; RCM—restrictive cardiomyopathy; DCM—dilated cardiomyopathy; ARVC—right ventricle arrhythmogenic cardiomyopathy; LVNC—left ventricular noncompaction cardiomyopathy.

**Table 2:** Summary of commonly used pediatric HF drugs and doses.

ACE inhibitors	
Captopril	0.5–2 mg/kg/dose 2–5 times/day (max 25 mg/dose)
Enalapril	0.1–0.5 mg/kg/day (max 20 mg/dose)
Lisinopril	0.05–2 mg/kg/die once or twice/day
**Angiotensin receptor blockersc (ARB)**	
Losartan	0.5–2 mg/kg/day
**Beta-blockers**	
**B1 selective**	
Bisoprolol	0.05–0.2 mg/kg/day 1–2 times/day
Metoprolol	0.1 mg/kg dose 2 times/day (max 1 mg/kg dose)
**B1 + B2 selective**	
Propranolol	0.2–0.5 mg/kg/dose 3 times/day
Carvedilol	0.1 mg/kg 2 times/day
**Diuretics**	
Furosemide	0.5–2 mg/kg/die
Spironolacton	0.5–2 mg/kg/die
Hydrochlorothiazide	0.5–1 mg/kg/die

**Table 3:** Summary of the clinical trials on new drugs for HF in pediatric and adult populations.

Drugs	Population	Title	Key Findings	Dose	Approval
Ivabradine	Adult	Ivabradine and outcomes in chronic heart failure (SHIFT): a randomized placebo-controlled study [18]	*N* = 6558 patients symptomatic for heart failure and an LVEF of 35% RCT with placebo, reduction of CV death, or hospital admission for worsening heart failure.	5 mg twice per day. Increase the dose after 2 weeks to 7.5 mg × 2/d	EMA approval in 2011
	Pediatric	Ivabradine in children with DCM and symptomatic chronic HF trial: a randomized, double-blind, placebo-controlled trial with 12-months follow-up [21]	*N* = 116; Ivabradine safely reduced the resting HR of children with chronic HF and CMD and an improvement in ejection fraction, functional class, and NT-pro BNP was noted.	Dose: 0.02–0.05 mg/kg × 2v/die < 40 kg, >40 Kg 2.5 mg × 2/die	FDA approval in 2019 EMA off label
Sacubitril/Valsartan		Angiotensin–Neprilysin Inhibition versus Enalapril in Heart Failure (PARADIGM-HF) [22]	*N* = 8442 patients with class NYHA II, III, or IV and FE ≤ 40% to receive either S/V (at a dose of 200 mg twice daily) or enalapril (at a dose of 10 mg twice daily), in addition to recommended therapy. S/V was superior to enalapril in reducing the risks of death and hospitalization for heart failure.	24/26 mg, 49/51 mg, and target dose 97/103 mg twice a day	FDA and EMA approval
	Pediatric	Design for the sacubitril/valsartan (LCZ696) compared with enalapril study of pediatric patients with heart failure due to systemic left ventricle systolic dysfunction (PANORAMA-HF study) [32]	*N*= 393 patients waiting for all subjects to complete the 52 weeks of therapy before performing data analysis.	<40 kg, the starting dose is 1.6 mg/kg of the combined amount of both valsartan and sacubitril. Aum every 2 weeks upward from 2.3 mg/kg up to a max dose of 3.1 mg/kg based on tolerance.	In October 2019, the FDA patients with symptomatic heart failure with LV systolic dysfunction, >1-year-old
Dapaglifozin	Adult	Dapagliflozin in Patients with Heart Failure and Reduced Ejection Fraction [23] Effect of Empagliflozin on the Clinical Stability of Patients With Heart Failure and a Reduced Ejection Fraction [24]	*N* = 4744 patients with HFrEF, dapagliflozin reduces the risk of worsening heart failure or death from CV causes compared to placebo *N* = 3730 patients with HFrEF double-blind treatment with placebo or empagliflozin, reduced the risk and total number of inpatient and outpatient worsening HF events, with benefits seen early after 12 days	10 mg once daily	FDA and EMA approval
	Pediatric	Early Clinical Experience with Dapagliflozin in Children with Heart Failure [26]	*N* = 38 patients dapagliflozin was added to the HF regimen, and after 312 days of therapy, LVEF increased significantly from 32 to 37.2%	0.1–0.2 mg/kg once daily (max 10 mg)	No approval
Omecamtiv	Adult	Cardiac Myosin Activation with Omecamtiv Mecarbil in Systolic Heart Failure [27]	*N* = 8256 patients with HFrEF receive omecamtiv mecarbil or a placebo, in addition to standard heart-failure therapy, reduce the incidence of a composite of a heart-failure event or death from cardiovascular causes	25 mg, 37.5 mg, or 50 mg twice daily	No approval
	Pediatric	No data	No data	No data	No data
Vericiguat	Adult	Vericiguat in Patients with Heart Failure and Reduced Ejection Fraction (VICTORIA trial) [29]	*N* = 5050 patients with HFrEF, vericiguat reduced risk of cardiovascular death, all-cause death, and HF hospitalization (phase 3)	starting dose 2.5 mg orally once daily with food. Double the dose every 2 weeks to reach the target e dose of 10 mg once daily	FDA and EMA approval in 2021
	Pediatric	No data	No data	No data	No data

## Data Availability

Not applicable
